# The Known and Unknown: Investigating the Carcinogenic Potential of Plastic Additives

**DOI:** 10.1021/acs.est.3c06840

**Published:** 2024-06-03

**Authors:** Sophia Vincoff, Beatrice Schleupner, Jasmine Santos, Margaret Morrison, Newland Zhang, Meagan M. Dunphy-Daly, William C. Eward, Andrew J. Armstrong, Zoie Diana, Jason A. Somarelli

**Affiliations:** † Department of Medicine and the Duke Cancer Institute Center for Prostate and Urologic Cancer, Duke University Medical Center, Durham, North Carolina 27710, United States; ‡ Department of Orthopaedics, Duke University Medical Center, Durham, North Carolina 27710, United States; § Nicholas School of the Environment, Duke University, Durham, North Carolina 27710, United States; ∥ Division of Marine Science and Conservation, Nicholas School of the Environment, Duke University Marine Laboratory, Duke University, Beaufort, North Carolina 28516, United States; ⊥ Department of Ecology and Evolutionary Biology, University of Toronto, 25 Wilcocks Street, Toronto, Ontario M5S3B2, Canada

**Keywords:** cancer, carcinogens, plasticizers, polymer, gene expression

## Abstract

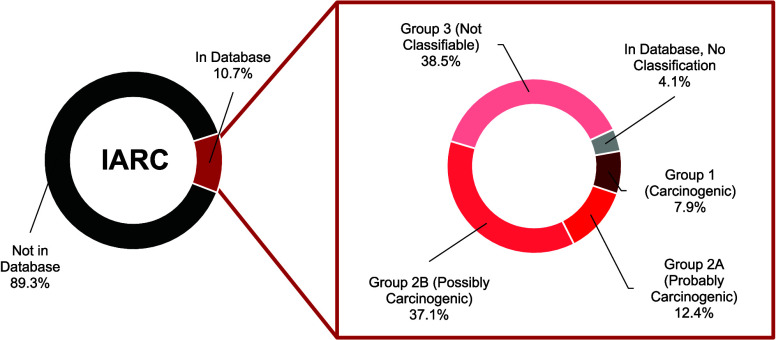

Microplastics are routinely ingested and inhaled by humans and other organisms. Despite the frequency of plastic exposure, little is known about its health consequences. Of particular concern are plastic additives—chemical compounds that are intentionally or unintentionally added to plastics to improve functionality or as residual components of plastic production. Additives are often loosely bound to the plastic polymer and may be released during plastic exposures. To better understand the health effects of plastic additives, we performed a comprehensive literature search to compile a list of 2,712 known plastic additives. Then, we performed an integrated toxicogenomic analysis of these additives, utilizing cancer classifications and carcinogenic expression pathways as a primary focus. Screening these substances across two chemical databases revealed two key observations: (1) over 150 plastic additives have known carcinogenicity and (2) the majority (∼90%) of plastic additives lack data on carcinogenic end points. Analyses of additive usage patterns pinpointed specific polymers, functions, and products in which carcinogenic additives reside. Based on published chemical–gene interactions, both carcinogenic additives and additives with unknown carcinogenicity impacted similar biological pathways. The predominant pathways involved DNA damage, apoptosis, the immune response, viral diseases, and cancer. This study underscores the urgent need for a systematic and comprehensive carcinogenicity assessment of plastic additives and regulatory responses to mitigate the potential health risks of plastic exposure.

## Abstract 2

Little is known regarding the carcinogenic potential of plastic additives. This research provides a toxicogenomics approach to screen additives for cancer-related dysregulation of gene expression and identify additives of potential concern.

## Introduction

Plastics, over the last half-century, have established a worldwide presence in nearly all societies and are widely detectable as pollutants in the environment. Demand for plastics has skyrocketed since the 1950s due to their inexpensive, strong, durable, and lightweight properties.^1^ Between 1950 and 2015, 8.3 billion metric tons of plastic products were created worldwide, 75% of which became waste.^2^ Global plastic production is expected to double by 2040.^3−5^ Despite benefits to societal health and safety, as well as a global market value of USD 569.9 billion in 2019,^4,6^ there is growing concern about plastic pollution and its impact on human and organismal health.^7−13^

Humans regularly interact with plastics through food packaging, clothing, toiletries, household items, furniture, automotive parts, medical equipment, electronics, toys, and office supplies.^14^ All plastics experience weathering, leading to the release of microplastics (1 μm to 5 mm) and nanoplastics (<1 μm).^15−17^ While initial human interactions with plastics are typically by choice, the ubiquitous persistence of plastics in the environment means that many subsequent exposures are involuntary. For instance, humans are routinely exposed to plastic particles through respiratory, oral, and dermal routes.^18−20^ As a result, plastic has been detected in human tissue and secretions, such as the lungs, colon, breast milk, and placenta.^21−24^ Given the widespread presence of plastics and microplastics in the environment and in human bodies, there is an urgent need to determine the health impacts of plastics. At present, there is far more information regarding exposure to individual polymers or specific plastics than there is about the lifetime exposure to all plastics.^25^

The risks of plastic exposure cannot be assessed without first acknowledging that plastics are not pure substances but rather complex mixtures of polymers along with dozens to thousands of chemical compounds broadly categorized as additives.^26,27^ Common additives used for performance enhancement include plasticizers, flame retardants, heat and light stabilizers, antioxidants, lubricants, pigments, antistatic agents, slip agents, biocides, and thermal stabilizers.^28^ Plastics also contain nonintentionally added substances from manufacturing, such as residual monomers, byproducts, and contaminants.^14^ During and after plastic usage, additional substances are adsorbed from the environment,^29^ such as polycyclic aromatic hydrocarbons or alkylphenols.^30^

Whether intentionally incorporated or not, plastic additives have the potential to leach from plastics and contaminate soil, air, water, food, and human bodies.^31^ Additives can comprise a sizable mass fraction of a plastic polymer,^32^ such as plasticizers, which can comprise up to 70% of the weight (w/w) of some polymers.^31^ Plastic additives have been detected in biota and throughout the environment, including in the tissues of shellfish,^33^ fish,^34,35^ seabirds,^36^ and marine mammals,^37^ underscoring the need to elucidate the impacts of these chemicals on organismal health.

Previous studies have identified many commonly used plastic additives, including those often used in food-contact products as well as those that should be further studied for their potential impacts on organismal health.^28,31^ Other studies have begun to identify the additives used in particular sectors of the plastic industry (e.g., packaging), but thousands of additives remain uncharacterized.^14,28,31^ With the increasing exposure to micro- and nanoplastics throughout the world, it is critical to understand the potential carcinogenic hazards of plastic additives.

Plastic additives have been demonstrated to impact multiple biological processes, such as metabolism, adipogenesis, and endocrine signaling. Among these impacts, both plastic polymers and their additives have been implicated in cancer.^18,28,29,38,39^ Cancer can have broad-ranging effects across scales of biological organization, from DNA-level and cellular alterations to population-level impacts.^40,41^ Microplastics have been associated with endocrine-related cancers, biliary tract cancer, hepatocellular carcinoma, and pancreatic cancer.^18^ For example, polycyclic aromatic hydrocarbons in polystyrene (PS) and compounds such as carbon black and legacy flame retardants in recycled plastic are often classified as carcinogenic.^28^ Similarly, heavy metals, many of which are carcinogens, are often used as colorants, stabilizers, and other functional additives.^29^ Although there exists data regarding the carcinogenicity of particular plastic additives, the literature lacks sufficient information regarding additive mixtures and environmentally relevant exposures to these additives.

To pinpoint potential additives of concern, we developed an analytical pipeline to identify chemical additives with known toxicological end points, determine impacts on gene expression pathways, and identify potential polymers and products in which these additives may reside. This can be done for single additives or combinations of additives. To do this, we curated a list of over 2,700 additives through a literature search of three databases. By querying two public chemical registries, we identified those additives with known and unknown carcinogenic potential. Using a toxicogenomics approach, we assessed the potential mechanisms of carcinogenicity and identified enriched pathways for all of the additives. The majority of our additives (∼90%) were unclassified as to their carcinogenicity in two major registries, due to either a lack of toxicological data or no public concern over the danger of the chemical. However, of the 229 unclassified additives with enough published gene expression data for analysis, a substantial portion (80.3%) induced pathways related to cancer and cancer-like phenotypes. Together, these analyses demonstrate a dearth of public knowledge regarding plastic additive carcinogenicity and pinpoint the need for a comprehensive experimental framework to determine the toxicological effects of plastic additives.

## Methods

### Analytical Workflow

**Figure 1:**
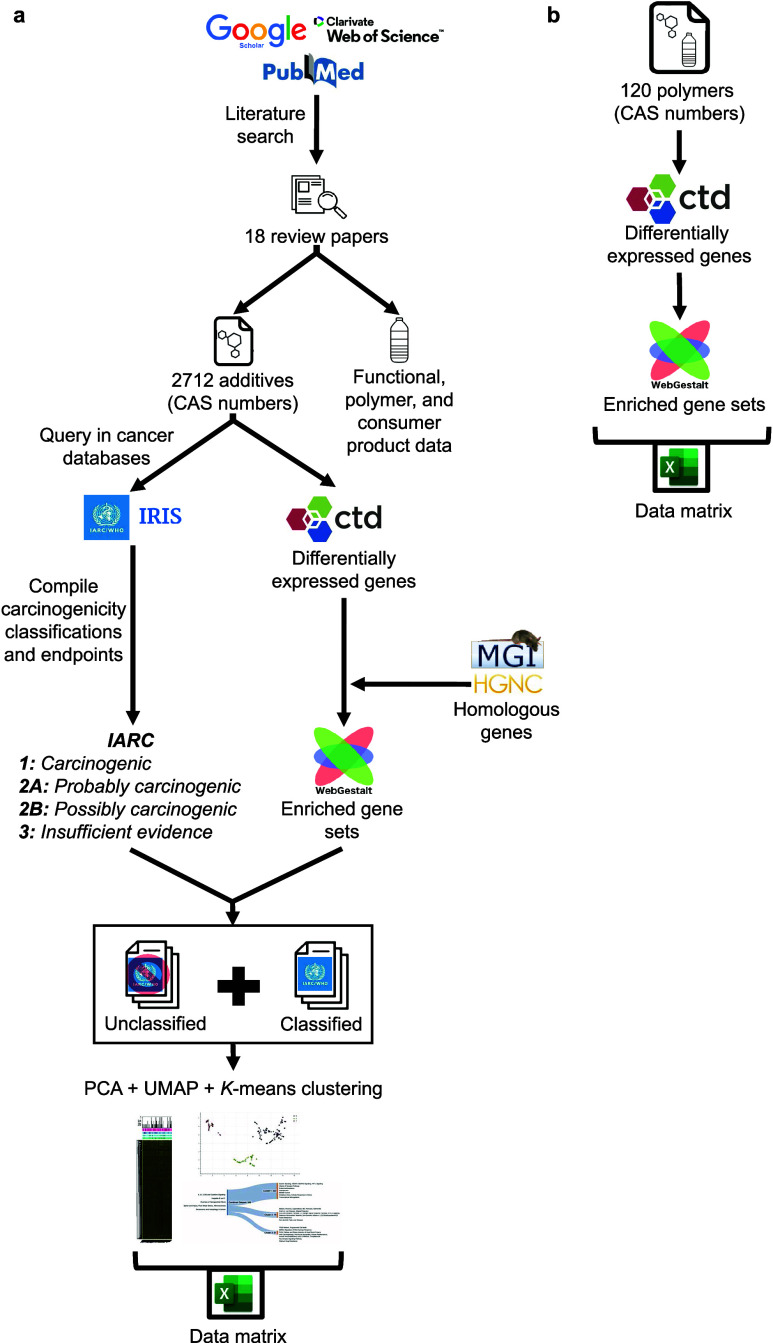
Resulting methodological workflow to analyze the carcinogenicity and gene expression patterns of (a) plastic additives and (b) polymers.

We developed an analytical workflow consisting of the following steps: (1) literature-based review and identification of plastic additives, (2) characterization of additive coverage in public cancer databases, and (3) integrated analysis of gene expression and usage data and cross-group comparisons. An *additive* is defined herein as any substance known to be added during the manufacturing process and/or detectable in the final polymer. Unexpected additives in the final polymer may be unintentionally added substances during manufacturing or substances that adsorbed from the environment during and after use. Any chemical in a polymer could theoretically leach out and cause harmful health effects. Therefore, we deem it critical to include any chemical to which a human might be exposed when ingesting or contacting plastic. A parallel analysis was also conducted on polymers (e.g., polyethylene, polystyrene, polyurethane) to compare the extent of knowledge on additives vs polymers. Figure 1 provides an outline of the bioinformatics workflow for the project.

### Literature-Based Review and Identification of Plastic Additives

To assemble a comprehensive list of plastic additives, we performed a literature review in Google Scholar, Clarivate Web of Science, and PubMed. In each database, targeted search strings were used to select peer-reviewed review articles containing lists of plastic additives (Table S1). Additives from each article were collected by their CAS numbers, assuming a one-to-one mapping of the CAS number to substance. When CAS numbers were not available within the source publication, the CAS number was retrieved from PubChem based on the chemical name. Several articles provided measures of confidence regarding the usage and presence of an additive; in these cases, only high-confidence additives were extracted. For example, Wiesinger et al.^14^ developed a weighted scoring metric to assess whether each of 2,486 additives of potential concern was truly present in plastic, ultimately assigning each chemical “high”, “medium”, or “low” confidence. The score considered the information origin, outlet control, and identification method. If any additive came from multiple primary sources, the highest of individual scores was selected to represent the chemical. We collected only the 1,985 chemicals with high confidence of presence in plastic. We chose to include plastic additives from publications that have already scraped and filtered primary sources. By using a meta-analysis of 18 publications, we were able to crosscheck for plastic additives within multiple articles. After seven articles were searched, the unique additive contribution per article began to diminish, plateauing at zero by the 14^th^ paper (Figure S1). In total, 18 articles produced 2,712 unique additives. This does not rule out the possibility of a new database emerging in the future but suggests that the data set herein is comprehensive at the present time.

Sixteen papers compiled from the literature review included information about the function or purpose (e.g., plasticizer, flame retardant), polymer usage (e.g., PET, PVA, PVC), and/or product usage (industry or consumer product, e.g., construction material, electronics, toys, textiles) of each additive. These data were manually collected in Excel and compiled using the pandas Python package.^42,43^ Ambiguous terms were included in all potential categories (e.g., when “adhesive” was found in a combined column of functions and products, it was recorded in both the Function and Product columns of our database). Table S2 contains results for each additive; results organized by polymer, product, and function are provided in Tables S3–S5. To aid in interpretability and analysis, product strings were also grouped into categories using search strings (Table S6). Both positive and negative search strings were used to avoid erroneous categorizations. For example, in the clothing category, “tablecloth” would be included under the “cloth” positive search string; therefore, “tablecloth” was added as a negative search string.

Polymer names and acronyms were collected from 18 literature review papers and other peer-reviewed publications from our literature review of the field. They were mapped to CAS identifiers by using PubChem. Alternate CAS numbers (if applicable) were retained and stored in a separate list from the primary number. There was no direct 1:1 mapping between any of the following variables: the full chemical name, polymer acronym, and CAS number. To conduct gene expression analysis, all chemicals under the same CAS number were grouped together.

### Characterization of Additive Coverage in Public Cancer Databases

To evaluate the extent of accessible documentation on plastic additive carcinogenicity, two publicly available databases were selected: IRIS (Integrated Risk Information System from the U.S. EPA) and IARC (International Agency for Research on Cancer). Databases were queried using R Statistical Software (v4.2.1)^44^ and the tidyverse package.^45^ The IRIS database contains 651 chemicals, some of which are duplicated and have different carcinogenicity classifications depending on the exposure route. If a single chemical was listed as carcinogenic and noncarcinogenic for different exposure routes, it was listed as carcinogenic for the analysis. As of September 2022, the IARC Database contained 1,101 total chemicals: 161 in Group 1 (carcinogenic to humans), 107 in Group 2A (probably carcinogenic to humans), 327 in Group 2B (possibly carcinogenic to humans), and 506 in Group 3 (inadequate evidence for carcinogenicity in humans). Any inconsistencies in IARC and IRIS classifications are provided in Table S7. The IARC database was selected as the standard for categorizing chemicals prior to further downstream bioinformatics analyses. We considered chemicals in Groups 1, 2A, and 2B *carcinogens* in our analysis. The grouping of *carcinogens* and Group 3 chemicals was referred to as *classified* because all of these chemicals are classified in IARC and have had their carcinogenic potential evaluated. Any chemical lacking an IARC category is considered *unclassified* and has not been annotated with respect to its carcinogenic potential in IARC.

### Integrated Analysis of Gene Expression and Usage Data

Gene expression and pathway enrichment data were collected from the Comparative Toxicogenomics Database (CTD), Mouse Genome Informatics (MGI), HUGO Gene Nomenclature Committee (HGNC) Comparison of Orthology Predictions (HCOP), and WebGestalt (WGA).

CTD is a public database sponsored by the National Institute of Environmental Health Sciences (NIEHS) which stores 50,048,577 toxicogenomic relationships. In this study, CTD was used to compile lists of human genes up- and downregulated by each chemical additive. The database provided relationships for 289 unclassified chemicals and 139 classified chemicals. When polymer CAS numbers were separately screened through CTD, 29 substances (15 unique CAS numbers) were found to up- or downregulate human genes according to published studies. On occasion, CTD labeled the interacting organism as human but erroneously provided a nonhuman GeneID. In these instances, the Mouse Genome Informatics Vertebrate Homology and HUGO Gene Nomenclature Committee Comparison of Orthology Predictions (HCOP) databases were queried to identify the corresponding human Entrez ID. If multiple human Entrez IDs were associated with one nonhuman homologue from CTD, all human matches were substituted for the homologue.

Using the gene lists from CTD, over-representation analysis (ORA) was performed in WebGestalt to predict pathway interactions for each additive (and polymer). For each substance, the ORA input was a single list combining all up- and downregulated genes. The WebGestaltR library was used to conduct batch ORA for all 428 additives and 15 polymers (polymers under the same CAS were grouped together). The PANTHER, Reactome, KEGG, Wikipathways, and Wikipathways Cancer pathway databases were queried with an FDR threshold of 25% (calculated with the Benjamini-Hochberg method), a minimum of 10 genes, and a maximum of 2,000 genes (default). Results were generated for seven polymers (46.67%), 120 classified chemicals (86.33%, 65% of which were carcinogens), and 229 unclassified chemicals (79.24%) (Tables S2 and S3). Even after homology correction, a small percentage of genes from CTD (<1%) remained unmappable in WebGestalt, but the majority of gene identifiers were recognized.

### Clustering and Cross-Group Comparisons

Dimensionality reduction and clustering were performed for each plastic additive in the Python programming language using sklearn.cluster.KMeans, sklearn.decomposition.PCA, and umap.umap_. A pairwise matrix of enrichment ratio (ER) for each plastic additive and pathway was constructed to facilitate weighted clustering on the pathway enrichment. ER is defined by the following formula: ER = overlap/expect, where expect = (input*size)/background_genes. Input refers to the number of genes submitted (the number of genes upregulated or downregulated by the plastic additive), size refers to the number of genes in the pathway being considered, and background_genes is the size of the reference genome, 13,049 human genes (“genome” selection in WebGestalt). Principal component analysis (PCA) was performed to achieve 95% explained variance with minimal dimensionality. The resulting matrix was further reduced using uniform manifold approximation and projection (UMAP) to improve the clustering results.

Cluster quality was heavily dependent on the two nondeterministic algorithms in this workflow: UMAP and *k*-means. Running *k*-means after applying default UMAP parameters (n_neighbors = 40, n_components = 2, min_dist = 0.3) was not sufficient for any *k*, producing silhouette scores with low and sometimes negative values. Silhouette scores below zero indicate that elements have been assigned to the wrong clusters; scores near zero indicate that clusters overlap; and scores near 1 indicate that most elements cluster more closely within their assigned cluster than other clusters. To ensure high-quality clusters, a minimum silhouette score of 0.70 was selected. UMAP’s n_neighbors parameter was tested at all integer values between 2 and 20 inclusive, while min_dist was tested at values 0.0, 0.1, 0.25, 0.5, 0.8, and 0.99. We carried out *k*-means for all *k* between 3 and 10, and the number of clusters producing the optimal silhouette score was selected. The random states for both UMAP and *k*-means were modulated between five different values to capture a broader range of possible results. This procedure was repeated for (1) all additives and all ERs and (2) only additives enriching at least one pathway with a cancer keyword substring [“cancer”, “carcin”, “metasta”, “tumor”] and the ERs for those pathways.

All subsequent analyses were performed on the three clusters made from the full data set. To determine subgroupings with similar cancer effects, Wikipathways Cancer pathways differentially enriched across a cluster above a certain standard deviation threshold (10, 10, and 20 for clusters 1, 2, and 3 respectively) were selected. Only additives enriching at least one of those pathways were retained. Enrichment ratios were scaled using the scale() function in R, and both additives and pathways were hierarchically clustered using ComplexHeatmap. Each pathway was manually assigned to one cancer-relation category (cancer type, cell cycle/proliferation, cell death/survival, DNA damage, immune, and metabolism) based on its most prominent effects according to Wikipathways and published literature.

To distinguish the most salient pathways for each cluster, a binary matrix was constructed to indicate whether each additive in the cluster enriched or did not enrich a particular pathway. The 50 pathways with the most additive associations were considered the central pathways for that cluster. “Uniquely enriched” pathways for a cluster do not appear in any other cluster’s top 50. “Highly enriched” pathways for a cluster have at least one ER ≥ 100.

### Inferring Overlapping Gene and Pathway Alterations Across Additive Groups

To investigate shared gene expression and pathway alteration patterns between additives of unknown carcinogenicity (Group 3 and unclassified) and confirmed carcinogens (Group 1), we calculated the number of overlapping upregulated genes, downregulated genes, and enriched pathways between each pair of chemicals and visualized the top pairings (Figure 5a–f). We also collected all pathways enriched by Group 1 additives and arranged Group 3 (Figure 5g) and unclassified (Figure 5h) additives according to their enrichment of these pathways.

## Results

This study resulted in a methodological workflow (Figure 1) to compile a list of plastic additives, investigate the carcinogenicity classifications of the additives, determine known impacts on gene expression, predict additives’ interference with human biological pathways, and group additives according to their predicted pathway effects (Table 1).^46^ An abbreviated parallel analysis was conducted on 280 reported polymer backbones, such as poly(vinyl chloride) and latex (Table S3). All collected data regarding additives can be found in Table S2.

**Table 1:** Snapshot of the Additives Database, Including IARC Category, Function, Polymer, Product, Gene Dysregulation, and Pathway Enrichment Data for an Example Additive (Unclassified Additive 900-95-8)^a^

CAS	Name	IARC Category	Function	Polymers	Products	Upregulated Genes (Symbol)	Total Upregulated Genes	Downregulated Genes (Symbol)	Total Downregulated Genes	Enriched Pathways	Total Enriched Pathways
900-95-8	phentin acetate	unclassified	antimicrobial, light stabilizer, lubricant, sealant, stabilizer	PUR, PUR foam, PVC	PVC flooring, adhesives, bags, beach balls, blinds, blister packaging, boards, bottles, car seats, containers, diapers, doors, ear protection equipment, gutters, paints, plastisol roof, rainwear, scotch brite, sealants, sewage pipes, shower curtains, vinyl wallpaper, water pipes, windows, wrapping films	AR, FOLH1	2	KLK3, NKX3-1, PMEPA1	3	Prostate Cancer_K, Pathways In Cancer_K, Rho GTPases Activate PKNs_R, Androgen Receptor Signaling Pathway_W, Activated PKN1 Stimulates Transcription of AR (Androgen Receptor) Regulated Genes KLK2 and KLK3_R, miRNA Regulation of Prostate Cancer Signaling Pathways_W	6

a The full data set is available in Table S2.

### Plastic Additives Include Multiple Known Carcinogens and Many with Unknown Cancer-Causing Potential

**Figure 2:**
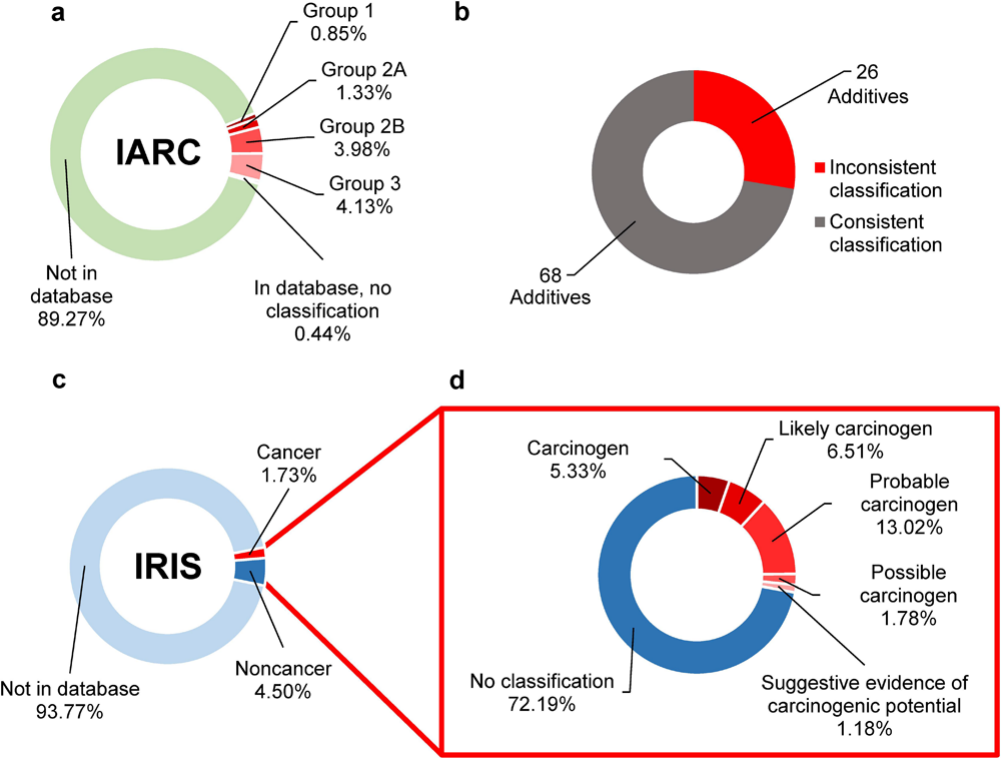
Majority of plastic additives are undocumented in toxicological databases. (a) Classification of the 2,712 plastic additives in the IARC database (Group 1: carcinogenic; Group 2A: probably carcinogenic; Group 2B: possibly carcinogenic; Group 3: not classifiable as to its carcinogenicity). (b) Number of consistent and inconsistent additive classifications in IRIS and IARC. (c) Classification of plastic additives in the IRIS database (Cancer = evidence of carcinogenicity or known carcinogen according to IRIS; Noncancer = chemical in the IRIS database, but no evidence suggesting carcinogenicity). (d) Detailed classifications within the “cancer” and “noncancer” categories in the IRIS database.

We first examined the presence and classifications of additives within the International Agency for Research on Cancer (IARC), which contained 1,101 chemicals at the time of our analysis (Figure 2a). A total of 2,421 additives (89.27%) were absent from IARC (Figure 2a). Among the 291 additives in the database, 12 (4.12%) had no classification, 112 (38.5%) had inadequate evidence for carcinogenicity and require more research (Group 3), 108 (37.1%) were possibly carcinogenic (Group 2B), 36 (12.4%) were probably carcinogenic (Group 2A), and 23 (7.9%) were carcinogenic (Group 1).

Cross-referencing all 2,712 additives in the IRIS database revealed 2,543 (93.77%) with no cancer-related data, 122 (4.50%) listed as noncancerous, and 47 (1.73%) listed as cancerous (Figure 2c). The IRIS database contained 651 chemicals at the time of our analysis. Of the 169 additives present in IRIS, 9 (5.33%) were carcinogens, 11 (6.51%) were likely carcinogens, 22 (13.02%) were probable carcinogens, 3 (1.78%) were possible carcinogens, and 2 (1.18%) had suggestive evidence of carcinogenicity (Figure 2d). Ninety-four additives were present in both the IARC and IRIS databases (Table S2), 26 of which had inconsistent cancer classifications between the two databases (Figure 2b, Table S7).

### Additive Usage Data Are Sparse

We next collected usage information for all plastic additives with available data (2,508 additives, 94.28%) from 18 review papers (Figure 1). In our analysis, *classified* additives are those assigned to Group 1, 2A, 2B, or 3 in IARC; *unclassified* additives are those absent from or unassigned in IARC; and *carcinogenic* additives are the subset of classified additives in Group 1, 2A, or 2B.

**Figure 3:**
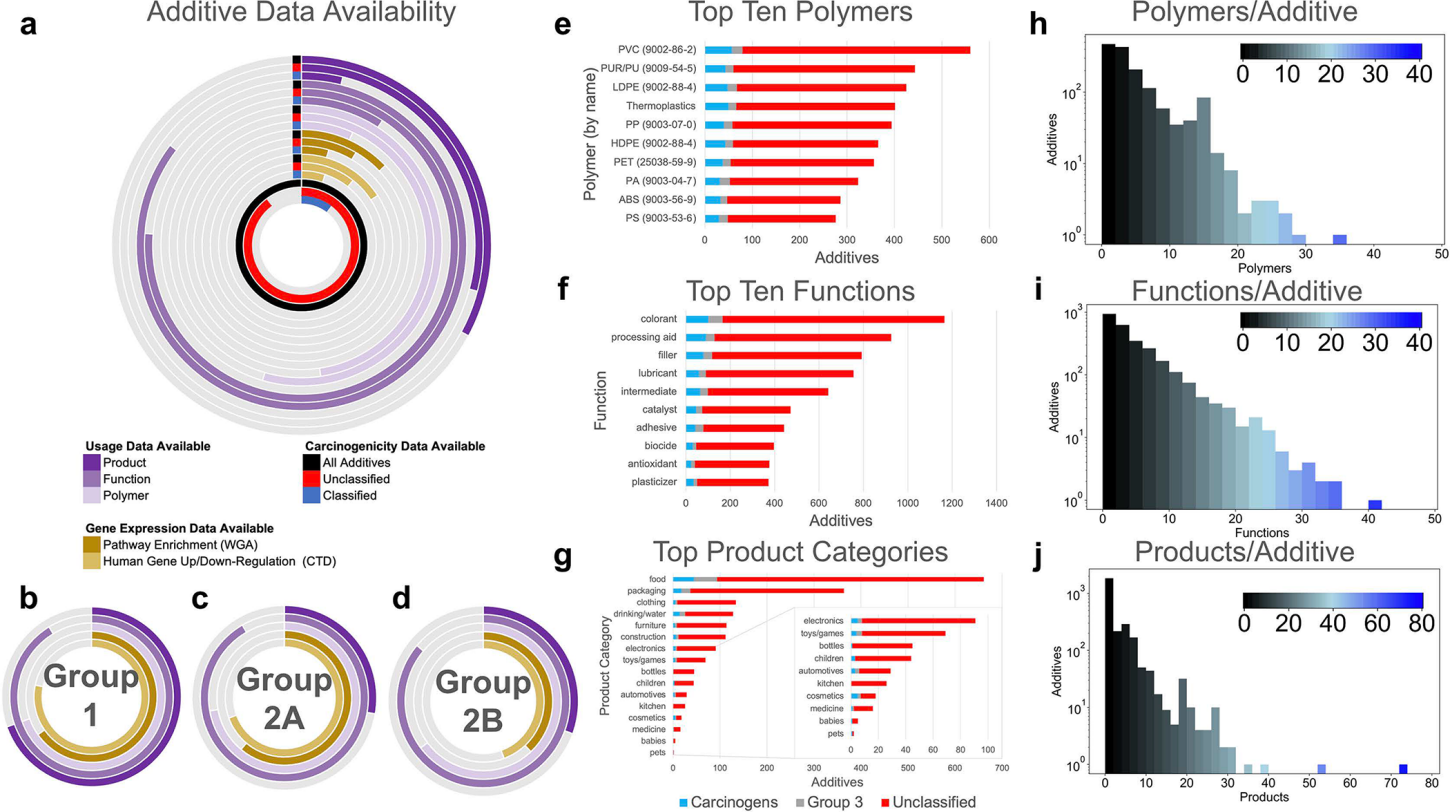
Data on usage and health effects for 2,712 plastic additives. (a) Product associations, polymer associations, effects on human gene expression, and possible disturbances to human gene networks for plastic additives. Dark-colored lines indicate the proportion of additives (out of 2,712) for which information is available. Rings marked or colored black represent all additives; red represents unclassified additives; blue represents classified additives. (b–d) Knowledge of additive carcinogenicity is associated with more knowledge of biological properties (Table S8). (e–g) The ten polymers, functions, and product categories with the most additive associations. Polymer associations were determined by Reference Name (Table S3). *LDPE and HDPE share the same CAS number. (h–j) Distributions of polymers/additive, functions/additive, and products/additive. Additives with no associations to polymers, functions, or products are not included in the histogram.

Analysis of usage data indicated 1,477 total additives (184 classified, 1,293 unclassified) associated with at least one polymer, 2,315 additives (248 classified, 2,067 unclassified) with at least one functional annotation, and 892 additives (104 classified, 788 unclassified) associated with at least one industrial or consumer product (Figure 3a, Tables S4 and S5). In total, 546 additives (84 classified and 462 unclassified) have usage data in all three categories (product, function, and polymer). Fewer than one-third of all carcinogenic additives are linked to industrial or consumer products (Figure 3b–d). Nine of the top ten polymers by additive association have traceable Chemical Abstracts Service Registry Numbers (CASRNs, or CAS numbers), and each is connected to hundreds of additives (Figure 3e). Nearly 400 additives are listed as components of “thermoplastics,” which encompass all plastics that become moldable at high temperatures and solidify upon cooling, including acrylic, polypropylene (PP), and polystyrene (PS). The top ten functions out of 167 unique function strings are reported in Figure 3f, with the top four (colorant, processing aid, filler, and lubricant) mapped to over 600 additives each. Sixteen product categories of interest were extracted from the product data by querying our database with specific search strings (Table S6). Food, packaging, and clothing-related products are associated with the most additives; medicine, babies, and pets are associated with the fewest (Figure 3g, Table S6).

Regarding the polymer data, we found that each additive is associated with 2.4 ± 3.9 polymers on average (Figure 3h), and >200 additives are associated with over 10 polymers each. Diethylhexl phthalate, a type 2B carcinogen (CAS = 117-81-7) has the maximum polymer associations (33) and 16 documented functions (Table S2). This additive was linked to diverse products including food packaging, plastic bags, medical equipment (*e.g.*, syringes, dialysis equipment, catheters, intravenous tubing, blood/dialysis bags, gaskets, implants, gloves), baby products (*e.g.*, pacifiers), plastic toys (*e.g.*, soft squeeze toys, balls, light sticks), bathroom products (*e.g.*, shower curtains, sanitary products), leisure products (*e.g.*, colored fishing floats, sports equipment), clothing (*e.g.*, raincoats), furniture (*e.g.*, floor tiles, furniture upholstery, tablecloths, flooring, wall coverings, wood coatings), and articles intended for pets.

The majority of additives are associated with up to five functions and products (Figure 3i,j), but several additives have dozens of matches in at least one usage category. Formaldehyde, a Type 1 carcinogen (CAS = 50-00-0), is the most functionally heavy, with 38 documented functions. This chemical also features 17 polymer associations and nine product associations, including food contact products, manufacturing container metals, and car seat stuffing. Similarly, butylated hydroxytulouene (CAS = 128-37-0; a class 3 chemical in IARC) and bisphenol A (80-05-7; a chemical unclassified in IARC) have very high numbers of both function and polymer associations (Table S2).

### Plastic Additives Impact Diverse Gene Expression Pathways

We used the up- and downregulated genes associated with all plastic additives in the Comparative Toxicogenomics Database (CTD) (18782832) as inputs for over-representation analyses in WebGestalt (WGA) (31114916). The most commonly upregulated genes by plastic additives include the tumor suppressor TP53; the proinflammatory cytokines C-X-C Motif Chemokine Ligand 8 (CXCL8, IL-8) and CXCL6 (IL-6); genes responsible for detoxification and metabolism of toxins, such as CYP1A1; and the cell cycle regulator, CDKN1A. The genes downregulated by the greatest number of additives included the apoptosis regulators, BCL2, BCL2L1, and BAX, and the cell adhesion molecule and epithelial lineage marker, E-cadherin (CDH1). Whether additives are classified or unclassified in regard to carcinogenicity, the reported effects on gene expression are similar (Table 2).

**Table 2:**
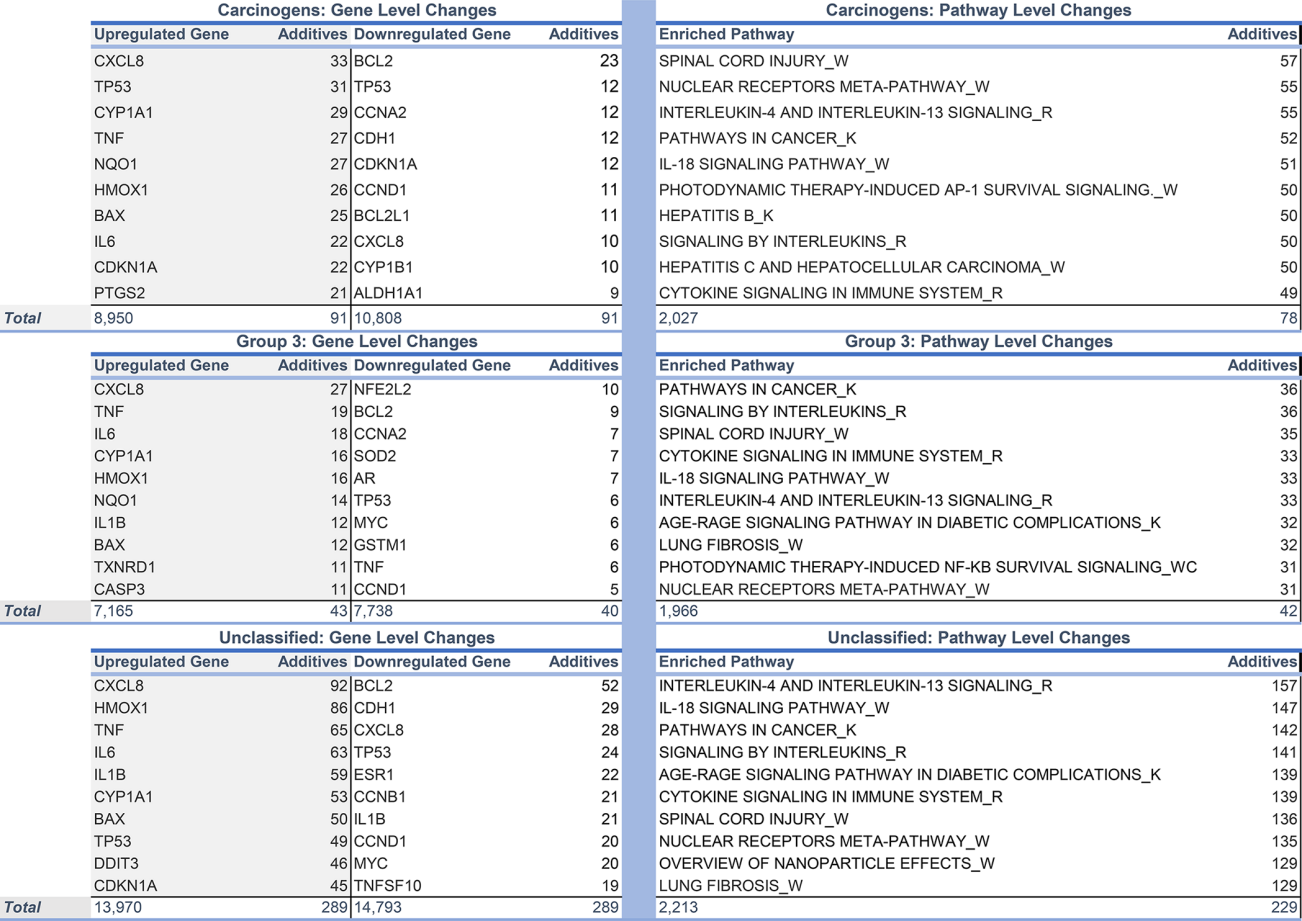
Top Up- and Downregulated Genes (Left), and Enriched Gene Sets (Right) for Different Groupings of Additives (Carcinogenic, Group 3, and Unclassified)

At the pathway level, carcinogenic and unclassified additives have similar impacts. Pathways altered by both carcinogens and unclassified additives include pathways in cancer and signaling by interleukins. However, unclassified additives, but not carcinogens, alter lung fibrosis and the AGE-RAGE signaling pathway in diabetic complications (Table 2).

Of the 2,712 additives, only 428 (15.78%, 139 classified, 289 unclassified) modulated human gene expression according to the CTD, and 349 additives (12.87%, 120 classified, 229 unclassified) contained enough gene interactions for over-representation analysis (Figure 3a). As IARC predictions intensified, from unclassified to Group 1, the number of papers documenting chemical–gene interactions increased (Figure 3a–d, Table S8). Group 1 carcinogens were found to have significantly more gene interaction data (*p* < 0.05) than any other group (Table S8).

### Based on Pathway Enrichment Ratios, Classified and Unclassified Additives Cluster into Three Unique Groups

**Figure 4:**
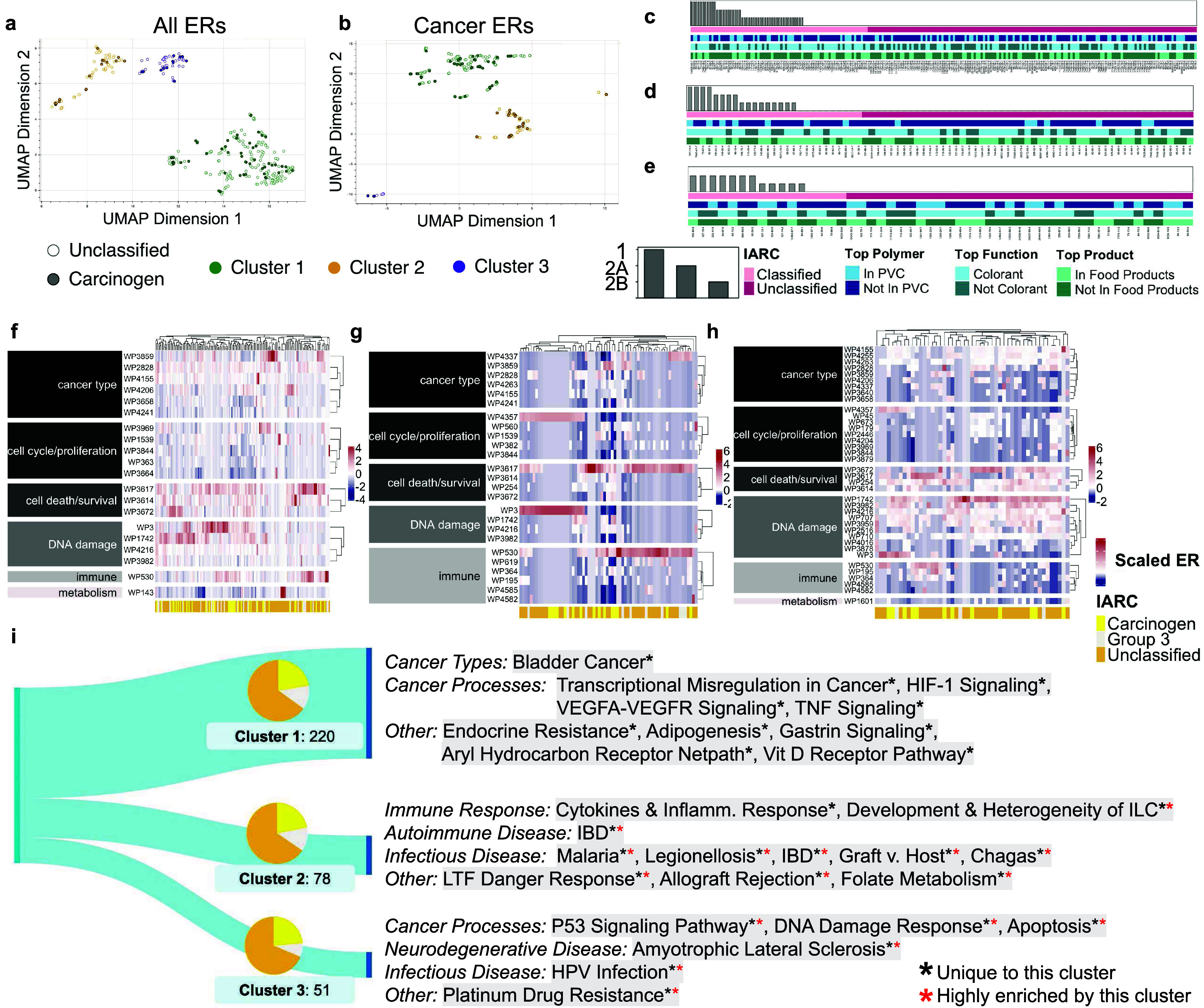
Carcinogens (probable, possible, or confirmed) and unclassified additives share similarities in their impacts on gene expression. When additives are clustered on their ERs for KEGG, Reactome, Wikipathways, and PANTHER gene sets, they form three distinct groups containing near-identical distributions of carcinogens, Group 3 additives (inadequate evidence for carcinogenicity in humans), and unclassified additives. (a) Three clusters (silhouette score = 0.86) encompass all additives, based on ERs for all 2,246 pathways. (b) Three clusters (silhouette score = 0.87) encompass additives enriching pathways with cancer keywords, based on ERs for those pathways only. (c–e) Clusters 1, 2, and 3 from panel a, respectively. Additives are sorted by IARC classification and display diverse usage patterns based on associations with the top polymer (PVC), function (colorant), and product category (food products). (f–h) Clusters 1, 2, and 3 from panel a, respectively. Each heatmap includes pathways from Wikipathways Cancer that are differentially enriched across the cluster. Pathways are divided by behavior and hierarchically clustered. Additives are hierarchically clustered by scaled ER and form subgroupings based on predicted cancer effects. (i) Sankey diagrams characterize each cluster from panel a, highlighting their uniquely and highly enriched gene sets. Pie charts show that clusters 1, 2, and 3 have near-identical distributions of carcinogenic, Group 3, and unclassified additives (although cluster 3 lacks any type 1 carcinogens).

We next used *K*-means and hierarchical clustering to visualize the relationships between additives at the pathway level (Figure 4). Pathway over-representation enrichment ratios (ERs) were used as input for the clustering. Clustering on all additives and all ERs produced silhouette scores, indicating that *k* = 3 clusters were optimal (Figure 4a). This clustering was largely unchanged when we analyzed subsets of data by similar pathway names (e.g., containing substrings of cancer keywords [“cancer”, “carcin”, “metasta”, “tumor”]) (Figure 4b), indicating that the clusters are well-separated. Notably, although each of the clusters are of different sizes, all clusters contain similarly proportioned mixtures of carcinogens (22–24%) and unclassified additives (65–69%) (Figure 4c–e), suggesting that carcinogenic and unclassified additives impact gene expression in similar ways (Cluster 1: 143 unclassified, 49 carcinogenic; Cluster 2: 51 unclassified,17 carcinogenic; Cluster 3: 35 unclassified, 12 carcinogenic). Additives within each cluster also exhibit diverse usage data. A mixture of Groups 1, 2A, 2B, and 3 and unclassified additives are present in PVC (the most common polymer), used as colorant (the most common function), and/or found in food products (the most common product category), but the specific proportions vary by cluster (Figure 4c–e).

To demonstrate how deeper connections between additives and cancer can be extracted from our data set, we sorted the additives within each cluster into subgroups with similar cancer effects. This was done through hierarchical clustering on the additives’ ERs for Wikipathways Cancer gene sets (Figure 4f–h). Each *k*-means cluster exhibits a unique profile. Much of Cluster 1 (Figure 4f) leans toward pathways impacting cell death/survival and DNA damage, with subgroups strongly impacting metabolic pathway WP143 (fatty acid beta-oxidation), immune pathway WP530 (cytokines and inflammatory response), and several cancer type pathways, specifically WP3859 (TGF-beta signaling in thyroid cells for epithelial-mesenchymal transition). Cluster 2 (Figure 4g) appears to be broken into three main segments: the first subgroup strongly enriching cell cycle/proliferation pathway WP4357 (NRF2-ARE regulation) and DNA damage pathway WP3 (transcriptional activation by NRF2 in response to phytochemicals); the second subgroup strongly enriching cell death/survival pathway WP3617 (Photodynamic therapy-induced NF-kB survival signaling) and slightly enriching cancer type pathway WP3859; and the third subgroup enriching cell death/survival pathway WP3617, immune pathway WP530, and cancer type pathway WP4337 (ncRNAs involved in STAT3 signaling in hepatocellular carcinoma). Cluster 3 (Figure 4h) enriches cell death/survival, DNA damage, and cancer type pathways nearly across the board, with subgroups displaying particular enrichment for cell cycle/proliferation pathway WP4357, cell death/survival pathways WP3672 (lncRNA-mediated mechanisms of therapeutic resistance) and WP3617, DNA damage pathways WP1742 (TP53 network) and WP3, and immune pathway WP530. Even after hierarchical clustering by cancer-related effects, carcinogens and unclassified additives are interspersed (Figure 4f–h).

Together, the affected pathways cover vast territory including DNA damage, apoptosis, immune response, viral diseases, and cancer. Many pathways are affected by chemicals in all three clusters. However, distinguishing features of each individual cluster can be found through their unique and/or highly enriched pathways (Figure 4i).

### Identifying Similarity in Gene Expression Impacts Across Additives

**Figure 5:**
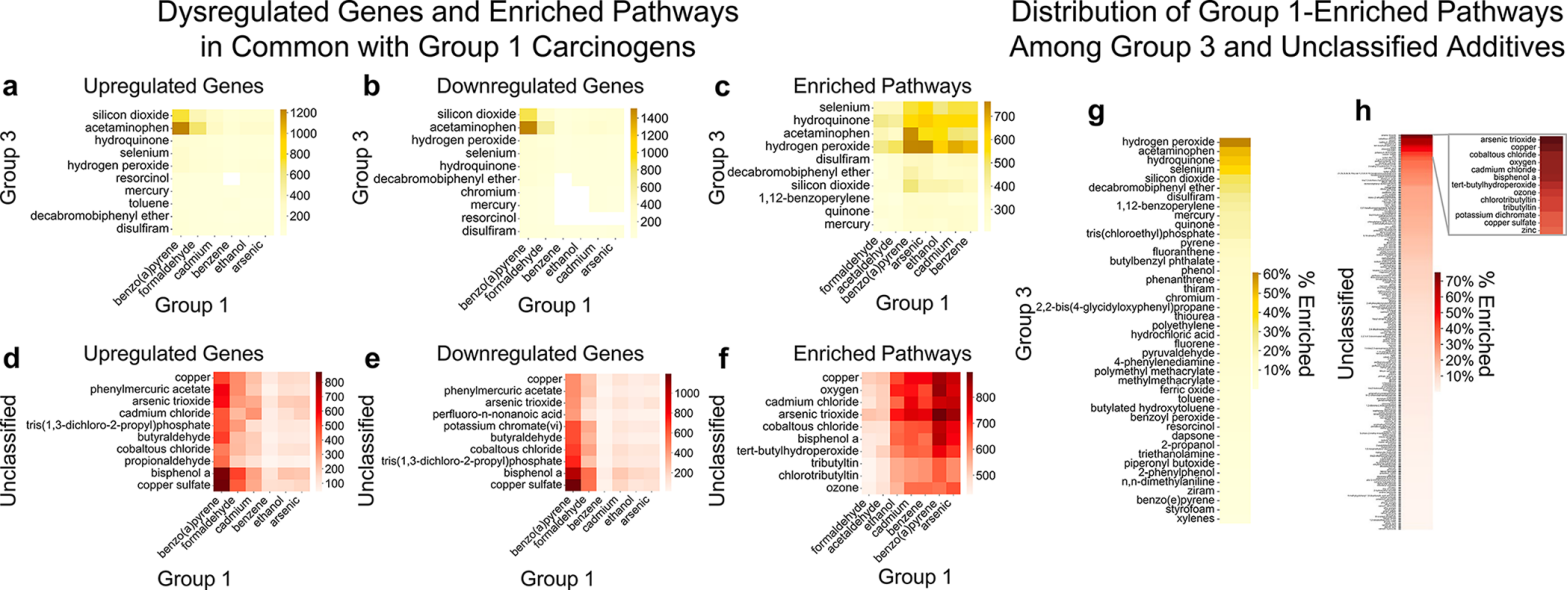
Comparison of carcinogenic additives with additives of unknown carcinogenicity. (a–f) The top ten Group 3 (a–c) and unclassified (d–f) additives with the greatest number of upregulated genes (a, d), downregulated genes (b, e), and pathways (c, f) in common with Group 1 carcinogens (*x*-axis). (g and h) Nonzero percentages of pathway overlap between Group 1 carcinogens and Group 3 additives (g) or unclassified additives (h).

Several additives with unknown carcinogenic potential group together in clusters, indicating a similar enrichment of biological pathways (Figure 4a). To better understand how Group 3 and unclassified additives may compare in their gene expression patterns to known carcinogens, we compared their upregulation, downregulation, and pathway enrichment patterns with those of carcinogens. We observed the greatest overlap between Group 3 (Figure 5a–c) and unclassified additives (Figure 5d–f) with confirmed (Group 1) carcinogens. The Group 1 additives with the most gene-level changes in expression were 50-32-8 (benzo(a)pyrene), 50-00-0 (formaldehyde), 7440-43-9 (cadmium), 71-43-2 (benzene), 64-17-5 (ethanol), and 7440-38-2 (arsenic). In Group 3, the following additives shared the most upregulated and downregulated genes with Group 1 carcinogens: 7631-86-9 (silicon dioxide), 103-90-2 (acetaminophen), 123-31-9 (hydroquinone), 7782-49-2 (selenium), 7722-84-1 (hydrogen peroxide), 108-46-3 (resorcinol), 7439-97-6 (mercury), 1163-19-5 (decabromobiphenyl ether), and 97-77-8 (disulfiram). Additionally, 108-88-3 (toluene) had significant upregulation overlap, and 7440-47-3 (chromium) had significant downregulation overlap (Figure 5a-b). The Group 1 additives with the greatest pathway enrichment were 50-32-8 (benzo(a)pyrene), 50-00-0 (formaldehyde), 7440-43-9 (cadmium), 71-43-2 (benzene), 64-17-5 (ethanol), 7440-38-2 (arsenic), and 75-07-0 (acetaldehyde). In Group 3, the ten additives sharing the most enriched pathways with these Group 1 carcinogens were 7782-49-2 (selenium), 123-31-9 (hydroquinone), 103-90-2 (acetaminophen), 7722-84-1 (hydrogen peroxide), 97-77-8 (disulfiram), 1163-19-5 (decabromobiphenyl ether), 7631-86-9 (silicon dioxide), 191-24-2 (1,12-benzoperylene), 106-54-1 (quinone), and 7439-97-6 (mercury) (Figure 5c).

Among unclassified chemicals, eight had the greatest overlap with Group 1 carcinogens in both upregulation and downregulation of gene expression, including 7440-50-8 (copper), 62-38-4 (phenylmercuric acetate), 1327-53-3 (arsenic trioxide), 13674-87-8 (tris(1,3-dichloro-2-propyl)phosphate), 123-72-8 (butyraldehyde), 7646-79-9 (cobaltous chloride), 80-05-7 (bisphenol A), and 7758-98-7 (copper sulfate). Additionally, 10108-64-2 (cadmium chloride) and 123-38-6 (propionaldehyde) had significant upregulation overlap, and 375-95-1 (perfluoro-n-nonanoic acid) and 7789-00-6 (potassium chromate (vi)) had significant downregulation overlap with Group 1 carcinogens (Figure 5d–e). The unclassified chemicals sharing the most enriched pathways with Group 1 chemicals were 7440-50-8 (copper), 7782-44-7 (oxygen), 10108-64-2 (cadmium chloride), 1327-53-3 (arsenic trioxide), 7646-79-9 (cobaltous chloride), 80-05-7 (bisphenol A), 75-91-2 (*tert*-butylhydroperoxide), 688-73-3 (tributyltin), 1461-22-9 (chlorotributyltin), and 10028-15-6 (ozone) (Figure 5f).

We also compared pathway alterations among Group 3 and unclassified additives with all Group 1 known carcinogens, which enriched a total of 1704 pathways (Figure 5g,h). Group 3 and unclassified additives shared consistent patterns in their enrichment of pathways enriched by Group 1 (Figure 5g,h). Together, these results pinpoint a subset of Group 3 and unclassified additives that share gene- and pathway-level changes with known carcinogens.

## Discussion

The pervasive nature of plastic and our frequent exposure to plastics has prompted increased attention to the potential harmful impacts of plastic on organismal health;^9^ however, many studies have focused on the influence of plastic polymers^47^ or particularly well-studied additives, such as bisphenol A.^48^ Far less is known about the comprehensive landscape of plastic additives and mixtures of additives, including their environmental fates, transport, and consequences for health and wellbeing. What little we know about additives is from studies on the additives in isolation, but these additives exist as complex mixtures of tens to hundreds of additives in a single plastic product (Figure 3e,g, Table S2), many of which exert widespread effects on gene expression (Figure 4f–i). Here, we created an analytical workflow to comprehensively characterize plastic additives for their potential carcinogenicity and impacts on gene expression. A striking observation from these analyses is the severe shortage of data on carcinogenic potential for hundreds of plastic additives (Figure 2a,c,d). The apparent lack of documentation and cross-verification among databases raises questions about the efficacy of current legislation and safety measures for plastics and plastic additives (Figure 2b). Almost 28% of additives that were documented in both IRIS and IARC had inconsistent cancer classifications (80.8% of which were listed as noncancer according to IRIS). These differences may be due to discrepancies in database annotation and maintenance.

Prior investigations have suggested that plastic ingestion may induce carcinogenesis. Studies in fish have shown that ingestion of microplastics induces hepatic inflammation^49^ and hepatic neoplasia.^50^ Plastic contains multiple known carcinogens, the most well-studied of which is bisphenol A.^51^ Notably, neither IARC nor IRIS listed bisphenol A as a carcinogen at the time of our analysis. At the cellular level, plastic exposure impacts numerous gene expression pathways linked to inflammatory signaling and cancer, including NF-κB,^52^ IL-6 ^53^, TNF alpha,^53^ and IL-8 (CXCL8).^54^ Consistent with these observations, our analyses revealed several dysregulations of these key genes by all classes of additives (Table S2). CXCL8 was the single most upregulated gene and was also often observed among the downregulated genes for carcinogens, unclassified additives, and Group 3 additives (Table 2). TNF alpha and IL6 were also top-upregulated and downregulated genes across the board (Table 2). NF-κB expression is upregulated and downregulated by multiple carcinogens (e.g., benzene upregulates and arsenic downregulates) as well as a number of unclassified additives.

Our investigation revealed numerous impacts of plastic additives on gene expression pathways, many of which are relevant to cancer, including pro-inflammatory signaling and oxidative stress pathways (Figure 4f–i, Tables 2 and S2). These effects on gene expression were exerted by both known carcinogens and additives for which the carcinogenic potential is unknown, and clustering by gene expression pathways revealed substantial overlap between carcinogenic additives and additives with unknown carcinogenic potential (Figure 4). Further, clusters of additives enriched many converging phenotypes, such as proliferation and antiapoptotic pathways, which may indicate greater carcinogenic risk (Figure 4h). Few pathways were consistently enriched across Groups 1, 2A, and 2B carcinogens. At the individual additive level, pathway enrichment results often reflect literature findings. For example, the unclassified additive alpha-pinene (80-56-8) exerts an antitumor effect by inducing G2/M cell cycle arrest.^55−57^ Consistent with this, alpha-pinene’s enriched pathways included G2/M Transition, G2/M DNA Damage Checkpoint, and three other G2/M pathways. Similarly, the Group 1 carcinogen trichloroethylene (79-01-6) enriched Prostate Cancer, Hepatocellular Carcinoma, and four similar pathways, which is consistent with epidemiological studies that linked this chemical to liver and prostate cancer in humans with significant exposures.^58^ The Group 2A carcinogen dimethylformamide (68-12-2) induces apoptosis in liver cells through the p53 pathway and maintains a redox status imbalance.^59^ Consistent with these findings, dimethylformamide enriched 13 apoptosis-related pathways (e.g., Apoptosis), 16 TP53/p53-related pathways (e.g., p53 Pathway), and oxidative stress pathways (e.g., Oxidative Stress Induced Senescence).

This study provides a platform to pinpoint plastic products that harbor mixtures of additives with known consequences on gene expression that may impact human health. Here, we have used existing toxicogenomic data to determine the carcinogenic pathways most likely to be impacted by plastic additives. The substantial clustering of gene expression pathways (Figure 4a,b) produced by carcinogenic, Group 3, and unclassified additives suggests that unclassified and Group 3 additives share gene expression patterns with known carcinogens, underscoring the need for further testing of these additives in toxicological analyses. These analyses may help researchers and policymakers to identify and prioritize the populations and products that contain mixtures of additives with the greatest potential for harm. Although the question of how to effectively regulate plastic additives remains extremely complex, this study provides a bioinformatic tool for screening 90% of additives that previously lacked data on carcinogenic potential. For example, we found 25 additives (including seven known carcinogens and 14 unclassified additives) associated with plastics in construction materials that activate colorectal and gastric cancer pathways. Consistent with this, construction workers are at an enhanced risk for multiple cancer types, including esophageal, colorectal, gastric, and testicular cancer.^60^ Whether these risks are associated with plastic additives requires further prospective interventional studies; however, our analysis provides a framework for identifying potential susceptible populations and associated products for a follow-up study.

The analyses presented here have several limitations. There is an overall lack of transparency in the industrial literature regarding the presence of additives in common plastic polymers. Over 4,000 chemicals are estimated to be used in plastic food packaging, but our literature review documented only 2,712 additives—many of which lacked polymer and product data—across all applications.^28,61^ The key limitations in synthesizing usage data were (1) misspelled, miscategorized, and unclear terms in the original review papers and (2) ambiguity when hierarchies of terms were created (e.g., “food-contact plastics”). Spelling corrections and grouping were either performed or checked manually because programmatic strategies like regex strings were prone to errors in prior research.^14^ However, manual database curation will not be scalable as research on plastic increases. A standardized and transparent way of disclosing, tracking, and reporting additives’ functions, polymers, and products will be necessary for the longevity of a comprehensive database.

Our toxicogenomics analysis revealed the presence of multiple carcinogenic additives in numerous plastic products. Perhaps more striking, however, is the severe lack of information about the carcinogenic potential for the overwhelming majority of plastic additives. At the gene expression level, these unclassified additives impact many of the same pathways as those of known carcinogens. Collectively, these data underscore the critical need for a systematic study of plastic additives with a focus on additives that overlap in their gene expression patterns with known carcinogens. We propose a transdisciplinary approach in which researchers, legislators, and manufacturers collaborate to address the following key gaps in our knowledge: (1) developing comprehensive toxicological profiles for individual plastic additives and common additive mixtures in plastic products, (2) mapping all additives to their functions and end points, (3) determining the fate and transport for individual additives and mixtures of additives that are leached from the same products in standardized settings, (4) identifying toxicological synergies between groups of additives, and (5) identifying high-priority additives that should be removed or replaced to preserve plastic functionality. While instituting new plastic additive regulations is likely to be difficult and multifaceted, tools that can pinpoint additives of potential concern for interventions and mitigation may help narrow the search space for carcinogenic additive combinations and accelerate reformulation strategies. We hope that this analytical pipeline can help guide future steps to enable a world where the health risks of plastics are both publicly known and effectively reduced.

## Data Availability

All code will be made available upon request. See the Supporting Information for all data. See our GitHub page, https://github.com/sophievincoff/Plastic-Additives, for .csv files of each supplementary table.

## Supporting Information Available

Review papers used to gather plastic additives (Sheet S1); all data collected for each additive (Sheet S2); all data collected for each polymer (Sheet S3); all data collected for each product (Sheet S4); all data collected for each function (Sheet S5); product categories (Sheet S6); inconsistencies between IARC and IRIS (Sheet S7); information coverage for Group 1, 2A, 2B, 3, unclassified additives (Sheet S8) (XLSX)
Data collection details: literature review and extraction of plastic additives; additive data compilation; polymer data compilation; usage data organized by function, product, and product category; identification of inconsistent chemical classifications; identifying significant data coverage differences across IARC categories (PDF)
