# Supplementary material for: The Known and Unknown: Investigating the Carcinogenic Potential of Plastic Additives
Source: Environ Sci Technol. 2024 Jun 3;58(24):10445–57. doi: 10.1021/acs.est.3c06840 (PMC11191590; doi:10.1021/acs.est.3c06840)
Supplement: Supplementary file 2 — es3c06840_si_002.pdf [file es3c06840_si_002.pdf]

Supporting Information S2

Environmental, Science & Technology: 2024

The Known and Unknown: Investigating the Carcinogenic Potential of Plastic Additives

Sophia Vincoff[1], Beatrice Schleupner[2], Jasmine Santos[1], Margaret Morrison[3], Newland Zhang[1], Meagan M. Dunphy-Daly[4], William C. Eward[2], Andrew J. Armstrong[1], Zoie Diana[4,5], Jason A. Somarelli[1,3*]

1. Department of Medicine and the Duke Cancer Institute Center for Prostate and Urologic Cancer, Duke University Medical Center, Durham, North Carolina 27710, United States
2. Department of Orthopaedics, Duke University Medical Center, Durham, North Carolina 27710, United States
3. Nicholas School of the Environment, Duke University, Durham, North Carolina 27710, United States
4. Division of Marine Science and Conservation, Nicholas School of the Environment, Duke University Marine Laboratory, Duke University, Beaufort, North Carolina 28516, United States
5. Department of Ecology and Evolutionary Biology, University of Toronto, 25 Wilcocks Street, Toronto, Ontario M5S3B2, Canada

TABLE OF CONTENTS

LIST OF TABLES **3**
LIST OF FIGURES **4**
S1 DATA COLLECTION DETAILS **5**
- **S1.1 LITERATURE REVIEW AND EXTRACTION OF PLASTIC ADDITIVES** 5
  - ***Table S1:*** *Major sources for plastic additive collection* *5*
  - ***Figure S1:*** *Literature search for plastic additives.* *6*
- **S1.2 ADDITIVE DATA COMPILATION** 7
  - ***Table S2:*** *Compiled data for all 2,712 plastic additives* *8*
- **S1.3 POLYMER DATA COMPILATION** 9
  - ***Table S3:*** *Compiled data for all 280 polymers.* *10*
- **S1.4 USAGE DATA ORGANIZED BY FUNCTION, PRODUCT, AND PRODUCT CATEGORY** 11
  - ***Table S4:*** *Additive-product associations.* *11*
  - ***Table S5:*** *Additive-function associations.* *11*
  - ***Table S6:*** *Additive-product category associations.* *12*
- **S1.5: IDENTIFICATION OF INCONSISTENT CHEMICAL CLASSIFICATIONS** 13

Table S7: *Inconsistent chemical classifications between IARC and IRIS databases.* *13*
S1.6: IDENTIFYING SIGNIFICANT DATA COVERAGE DIFFERENCES ACROSS IARC CATEGORIES 14
Table S8: *Significant differences in data coverage between additives in IARC Group 1, 2A, 2B, 3, and unclassified.* *14*

List of Tables

Table S1: *Major sources for plastic additive collection.* S5
Table S2: *Compiled data for all 2,712 plastic additives.* S8
Table S3: *Compiled data for all 280 polymers* S10
Table S4: *Additive-product associations* S11
Table S5: *Additive-function associations.* S11
Table S6: *Additive-product category associations* S12
Table S7: *Inconsistent chemical classifications between IARC and IRIS databases.* S13
Table S8: *Significant differences in data coverage between additives in IARC Group 1, 2A, 2B, 3, and unclassified.* S14

List of Figures

Figure S1: *Literature search for plastic additives. S6*

S1 DATA COLLECTION DETAILS

S1.1 Literature review and extraction of plastic additives

Table S1: Major sources for plastic additive collection. Additives were pulled from 18 total review articles, each with clear justification of why the chemicals they reviewed could be considered plastic additives. Only the first 13 articles (listed below) contributed unique additives to our list. Details on all 18 review papers surveyed can be found in Sheet S1 in Supporting Information S1 and at *https://github.com/sophievincoff/Plastic-Additives/blob/main/TableS1.csv*.

Paper	Source	Search String	Total Additives	Unique Additives Contributed	Justification
Wiesinger *et al.* (2021)	PubMed	*(plastic[Title] OR plastics[Title]) AND (additive[Title] OR additives[Title] OR toxicology[Title] OR chemical[Title] OR chemicals[Title])*	1985	1985	*Substances of potential concern that are considered with high confidence to be plastic monomers, additives, or processing aids*
Groh *et al.* (2019)	PubMed	*(plastic[Title] OR plastics[Title]) AND (additive[Title] OR additives[Title] OR toxicology[Title] OR chemical[Title] OR chemicals[Title])*	906	510	*Chemicals likely to be associated with plastic packaging*
Lithner *et al.* (2011)	Google Scholar	*(plastic OR plastics) AND (additive OR additives OR chemical OR chemicals) AND (review OR overview OR comprehensive OR analysis OR assessment)*	297	94	*Literature search for additives needed to produce specific polymers*
Stenmarck *et al.* (2017)	Google Scholar	*(plastic OR plastics) AND (additive OR additives OR chemical OR chemicals) AND (review OR overview OR comprehensive OR analysis OR assessment)*	341	55	*Hazardous substances known to be used in the manufacturing of plastics*
Rani *et al.* (2015)	Google Scholar	*(plastic OR plastics) AND (additive OR additives OR chemical OR chemicals) AND (review OR overview OR comprehensive OR analysis OR assessment)*	60	33	*Chemicals with high frequency of detection in plastic polymer items and marine debris*
Hahladakis *et al.* (2018)	PubMed	*(plastic[Title] OR plastics[Title]) AND (additive[Title] OR additives[Title] OR toxicology[Title] OR chemical[Title] OR chemicals[Title])*	82	11	*Additives present in plastics*
Carmen (2021)	Google Scholar	*(plastic OR plastics) AND (additive OR additives OR chemical OR chemicals) AND (review OR overview OR comprehensive OR analysis OR assessment)*	48	8	*Most commonly used plastic additives in petroleum-based plastic material manufacturing*
Akoueson *et al.* (2021)	PubMed	*(plastic[Title] OR plastics[Title]) AND (additive[Title] OR additives[Title] OR toxicology[Title] OR chemical[Title] OR chemicals[Title])*	45	7	*Additives identified by studying plastic samples using pyrolysis-gas chromatography coupled with mass spectrometry*
Campanale *et al.* (2020)	Web of Science	*(plastic or plastics) AND (additives or additive)*	38	4	*Additives present in microplastics*
Wagner *et al.* (2020)	Google Scholar	*(plastic OR plastics) AND (additive OR additives OR chemical OR chemicals) AND (review OR overview OR comprehensive OR analysis OR assessment)*	10	2	*Substances of very high concern with literature indicating use in plastic*
Bang *et al.* (2012)	Web of Science	*(plastic or plastics) AND (additives or additive)*	9	1	*Endocrine-disrupting plastic ingredients used to make food packaging materials*
Ong *et al.* (2020)	Web of Science	*(plastic or plastics) AND (additives or additive)*	9	1	*Additives included during the manufacturing and processing of plastic packaging materials*
Turner (2018)	PubMed	*(plastic[Title] OR plastics[Title]) AND (additive[Title] OR additives[Title] OR toxicology[Title] OR chemical[Title] OR chemicals[Title])*	6	1	*Chemical elements determined by X-ray fluorescence spectrometry in sample categories of black plastic*

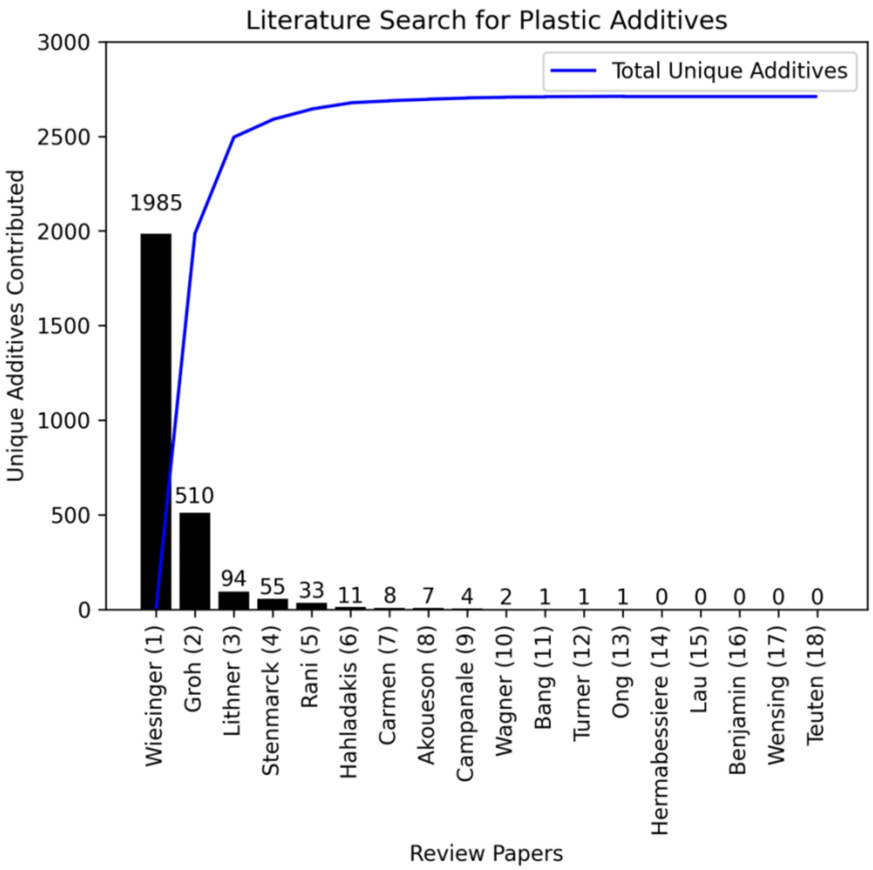

Figure S1: Literature search for plastic additives. As more review articles were considered, new additives plateaued**.** Eighteen articles were considered for plastic additive collection. The number of new additives contributed started to level out around the seventh review article, and hit zero by the fourteenth. In total, 2,712 unique plastic additives were found.

S1.2 Additive data compilation

Throughout our analysis, we compiled various types of data for each plastic additive. The resulting table (Table S2) contains the following columns: *'CAS', 'Name', 'Source', 'IARC Category', 'Functions', 'Total Functions', 'Polymers', 'Total Polymers', 'Polymers CAS', 'Products', 'Total Products', 'Enriched Pathways', 'Total Enriched Pathways', 'Upregulated Genes (Symbol)', 'Upregulated Genes (Entrez ID)', 'Total Upregulated Genes', 'Downregulated Genes (Symbol)', 'Downregulated Genes (Entrez ID)','Total Downregulated Genes', 'PubMedIDs on Gene Interactions', 'Total PubMedIDs on Gene Interactions', 'IRIS_assessment_type', 'IRIS_WOE_characterization', 'Cluster All ERs'*. Information for each category was retrieved as follows: *'CAS'*= CAS identifier, taken directly from the Source article or collected from PubMed using chemical name; *'Name'* = common chemical name for each CAS ID, taken from the Comparative Toxicogenomics Database (CTD) in the search for chemical-gene interactions; *'Source'* = the article(s) from which the additive was collected; *'IARC Category'* = the International Agency for Research on Cancer (IARC) classification (1, 2A, 2B, 3) or lack thereof, based on an IARC search in September 2022; *'Functions'* = all unique function strings associated with the chemical in its Source article(s); *'Total Functions'* = sum of strings in *Functions*; *'Polymers'* = all unique polymer strings associated with the chemical in its Source article(s); *'Total Polymers'* = sum of strings in *Polymers*; *'Polymers CAS'* = all unique CAS numbers associated with polymers in *Polymers*, pulled from PubChem based on polymer name; *'Products'* = all unique product strings associated with the chemical in its Source article(s); *'Total Products'* = sum of strings in *Products; 'Enriched Pathways*' = official descriptions of all enriched pathways from Over-Representation Analysis in WebGestalt, where suffix tags represent the source pathway database: _P = PANTHER, _R = Reactome, _K = KEGG, _W = Wikipathways, _WC = Wikipathways Cancer; *'Total Enriched Pathways'* = sum of strings in *Enriched Pathways*; *'Upregulated Genes (Symbol)'* = upregulated gene symbols directly provided by CTD; *'Upregulated Genes (Entrez ID)'* = upregulated gene Entrez IDs directly provided by CTD; *'Total Upregulated Genes'* = total unique upregulated gene symbols; *'Downregulated Genes (Symbol)'* = downregulated gene symbols directly provided by CTD; *'Downregulated Genes (Entrez ID)'* = downregulated gene Entrez IDs directly provided by CTD; *'Total Downregulated Genes'* = total unique downregulated gene symbols; *'PubMedIDs on Gene Interactions'* = PubMed IDs containing the chemical-gene interactions in the CTD, provided directly by the CTD; *'Total PubMedIDs on Gene Interactions'* = total unique IDs in *'PubMedIDs on Gene Interactions'*; *'IRIS_assessment_type'* = "Cancer" or "Noncancer" classification by Integrated Risk Information System (IRIS), if available; *'IRIS_WOE_characterization'* = Weight-of-the-Evidence (WOE) classification from IRIS, if available; *'Cluster All ERs'* = *k-means* cluster this additive was assigned (main text Fig. 4), or none if insufficient data for clustering.

Table S2: Compiled data for all 2,712 plastic additives. One example from each IARC Category (1, 2A, 2B, 3, or unclassified by IARC) is shown below; full information can be found in Sheet S2 in Supporting Information S1 and at *https://github.com/sophievincoff/Plastic-Additives/blob/main/TableS2.csv*.

CAS	Name	Source	IARC Category	Functions	Polymers	Polymers CAS	Products	Enriched Pathways (First 5)	Upregulated Genes (Symbol)	Downregulated Genes (Symbol)	PubMedIDs on Gene Interactions	IRIS Assessment Type	IRIS WOE Characterization	Cluster All ERs
101-14-4	methylenebis(chloroaniline)	Hahladakis et al. (2018), Wiesinger et al. (2021), Stenmarck et al. (2017), Sanchez (2021)	1	antioxidant, catalyst, chain extender, colorant, crosslinker, curing agent, heat stabilizer, intermediate, light stabilizer, lubricant, monomer, plasticizer, prepolymer, processing aid	PUR, PVC	68648-82-8, 9009-54-5		GLUTATHIONE METABOLISM_K, NRF2-ARE REGULATION_WC, FERROPTOSIS_K, FERROPTOSIS_W, NUCLEAR RECEPTORS META-PATHWAY_W	AKR1B10, GCLC, GCLM, GPAT3, LIF, SLC7A11, SQSTM1, SRXN1		26198647			2
62-53-3	aniline	Wiesinger et al. (2021)	2A		thermoplastics			ATF4 ACTIVATES GENES_R, PERK REGULATES GENE EXPRESSION_R, UNFOLDED PROTEIN RESPONSE (UPR)_R	ATF3, CXCL8, MPO	CYP19A1	27965148, 20199096, 16480277	Cancer	Probable Carcinogen	2
140-88-5	ethyl acrylate	Wiesinger et al. (2021), Groh et al. (2019)	2B	adhesive, biocide, colorant, filler, flame retardant, hardener, intermediate, lubricant, monomer, odor agent, process regulator, processing aid, surface treatment finishing agent, viscosity adjustor	HDPE, LDPE, PP	25087-34-7, 9002-88-4, 9003-07-0	adhesive, food contact, food-contact products, manufacturing plastics, manufacturing rubber	NRF2-ARE REGULATION_WC, FERROPTOSIS_K, FERROPTOSIS_W, PHYTOCHEMICAL ACTIVITY ON NRF2 TRANSCRIPTIONAL ACTIVATION_WC	AKR1B10, AKR1C2, ASF1A, FBXO30, GCLC, GCNT3, LIF	SLC7A11	26198647			2
1330-20-7	xylenes	Wiesinger et al. (2021), Groh et al. (2019), Lithner et al. (2011)	3	adhesive, anti-foaming agent, antimicrobial, antistatic agent, catalyst, colorant, dispersing agent, filler, flame retardant, hardener, lubricant, oxidizing agent, paint filler, plasticizer, sealing compound, softener, solvent, stabilizer, surface treatment agent, thickener, viscosity adjustor	PPE, PPO, TPU, thermoplastics	25322-69-4, 9009-54-5, 9041-80-9	adhesive, food contact, food-contact products, manufacturing container metals, manufacturing containers	NEUREXINS AND NEUROLIGINS_R, NEURONAL SYSTEM_R, PROTEIN-PROTEIN INTERACTIONS AT SYNAPSES_R	ACE, CARMIL3, CDCP2, CXCL8, DPT, ENTPD1, HSPA5, POLD4	BMP8B, CACNG8, CAMKV, CLCN4, CLIP2, CNTNAP3, DISC1, FEZ2, FLVCR1, FMR1, FN1, GLS2, GRM1, H3C15, IL1RAPL1, KLF4, MALAT1, NAMPT, NOTCH1, OTX1, PAPLN, RAB11FIP1, RP9P, SHANK1, SLITRK5, SULT4A1, SYT2, TP73, WASHC2C, WNT7B, YTHDF1	31734321, 12183065, 26018793, 33064461, 33438815	Noncancer	No Classification	1
100-51-6	benzyl alcohol	Groh et al. (2019)		adhesive, antistatic agent, cleaning agent, colorant, filler, hardener, lubricant, monomer, process regulator, solvent, stabilizer			adhesive, cleaning agents, food contact, food-contact products	INTERLEUKIN-4 AND INTERLEUKIN-13 SIGNALING_R, INTERLEUKIN-10 SIGNALING_R	IL1A, LIF		19878710			1

S1.3 Polymer data compilation

An abbreviated parallel analysis to that done on the plastic additives was performed for polymers. Gene expression data were collected and Over-Representation Analysis (ORA) was performed. Clustering was not performed, as there were not enough polymers with ORA data for clustering to yield fresh insights. The resulting table (Table S3) contains the following columns: '*Acronym', 'Name', 'Referenced As', 'Primary CAS', 'All CAS', 'Upregulated Genes (Symbol)', 'Upregulated Genes (Entrez ID)', 'Total Upregulated Genes', 'Downregulated Genes (Symbol)','Downregulated Genes (Entrez ID)', 'Total Downregulated Genes','PubMedIDs on Gene Interactions', 'Total PubMedIDs on Gene Interactions', 'Enriched Pathways', 'Total Enriched Pathways', 'Additives', 'Total Additives'*. Information for each category was retrieved as follows: *'Acronym'* = acronym for this chemical, taken directly from research article if available; *'Name'* = common name, taken directly from research article; *'Referenced As'* = name used to refer to this polymer in the document (either the *Acronym* or the *Name*), '*Primary CAS'* = primary CAS number used to refer to this chemical, taken either from literature article or PubChem; *'All CAS'* = primary and alternate CAS numbers, if available; *'Upregulated Genes (Symbol)'* = upregulated gene symbols directly provided by CTD; *'Upregulated Genes (Entrez ID)'* = upregulated gene Entrez IDs directly provided by CTD; *'Total Upregulated Genes'* = total unique upregulated gene symbols; *'Downregulated Genes (Symbol)'* = downregulated gene symbols directly provided by CTD; *'Downregulated Genes (Entrez ID)'* = downregulated gene Entrez IDs directly provided by CTD; *'Total Downregulated Genes'* = total unique downregulated gene symbols; *'PubMedIDs on Gene Interactions'* = PubMed IDs containing the chemical-gene interactions in the CTD, provided directly by the CTD; *'Total PubMedIDs on Gene Interactions'* = total unique IDs in *'PubMedIDs on Gene Interactions'*; *'Enriched Pathways*' = official descriptions of all enriched pathways from Over-Representation Analysis in WebGestalt, where suffix tags represent the source pathway database: _P = PANTHER, _R = Reactome, _K = KEGG, _W = Wikipathways, _WC = Wikipathways Cancer; *'Total Enriched Pathways'* = sum of strings in *Enriched Pathways; 'Additives'* = all plastic additives found associated with this polymer; *'Total Additives'* = sum of strings in *Additives*.

Table S3: Compiled data for all 280 polymers. A few representative example polymers are shown with abbreviated information for display (*e.g.*, only the first five Enriched Pathways are shown). Full information can be found in Sheet S3 in Supporting Information S1 and at *https://github.com/sophievincoff/Plastic-Additives/blob/main/TableS3.csv*.

Acronym	Name	Referenced As	Primary CAS	All CAS	Upregulated Genes (Symbol)	Upregulated Genes (Entrez ID)	Total Upregulated Genes	Downregulated Genes (Symbol) (first 5)	Downregulated Genes (Entrez ID) (first 5)	Total Downregulated Genes	PubMedIDs on Gene Interactions	Total PubMedIDs on Gene Interactions	Enriched Pathways (first 5)	Total Enriched Pathways	Additives (first 5)	Total Additives
XPS,EPS	expanded polystyrene	XPS,EPS	9003-53-6	9003-53-6	C3, C5, ITGAM, SELP	718, 727, 3684, 6403	4			0	19428933, 21123846	2	STAPHYLOCOCCUS AUREUS INFECTION_K, CELLS AND MOLECULES INVOLVED IN LOCAL ACUTE INFLAMMATORY RESPONSE_W, PERTUSSIS_K, COMPLEMENT AND COAGULATION CASCADES_K, HUMAN COMPLEMENT SYSTEM_W	21	91995-17-4, 8000-25-7, 50-32-8, 65996-93-2, 1163-19-5	113
	isoprene	isoprene	78-79-5	78-79-5	CCND1, CCNE1, CDH2, MIR423, MIR4284, SNAI2	595, 898, 1000, 494335, 100422948, 6591	6	CDH1, CDKN1A, CDKN1B, MIR1258, MIR1260A	999, 1026, 1027, 406911, 100302236	33	31765140, 30236600	2	MICRORNAS IN CANCER_K, MIRNAS INVOLVED IN DNA DAMAGE RESPONSE_W, MIRNA REGULATION OF DNA DAMAGE RESPONSE_W, METASTATIC BRAIN TUMOR_W, PARKINSONS DISEASE PATHWAY_W	110	120-78-5	1
PMMA	polymethylmethacrylate	PMMA	9011-14-7	9011-14-7	CASP9, IL6, TGFB1	842, 3569, 7040	3			0	34245821, 11403239	2	HEPATITIS C AND HEPATOCELLULAR CARCINOMA_W, VIRAL ACUTE MYOCARDITIS_W, HEPATITIS B_K, HEPATITIS B INFECTION_W, TUBERCULOSIS_K, CYTOKINES AND INFLAMMATORY RESPONSE_WC	141	108-88-3, 111-46-6, 115-96-8, 117-81-7, 123-28-4	22
PO	polyolefin	PO	89-25-8	89-25-8	FLT1, HIF1A, IL1B, NGF, VEGFA	2321, 3091, 3553, 4803, 7422	5			0	17889387, 19951488, 19954754, 24840734	4	RANKL/RANK (RECEPTOR ACTIVATOR OF NFKB (LIGAND)) SIGNALING PATHWAY_W, OSTEOBLAST SIGNALING_W, SUDDEN INFANT DEATH SYNDROME (SIDS) SUSCEPTIBILITY PATHWAYS_W, RUNX2 REGULATES OSTEOBLAST DIFFERENTIATION_R, RHEUMATOID ARTHRITIS_K	267	101-02-0, 101-77-9, 10191-41-0, 110-30-5, 112-84-5	65
PS	polystyrene	PS	9003-53-6	9003-53-6	C3, C5, ITGAM, SELP	718, 727, 3684, 6403	4			0	19428933, 21123846	2	STAPHYLOCOCCUS AUREUS INFECTION_K, CELLS AND MOLECULES INVOLVED IN LOCAL ACUTE INFLAMMATORY RESPONSE_W, PERTUSSIS_K, COMPLEMENT AND COAGULATION CASCADES_K, HUMAN COMPLEMENT SYSTEM_W	21	100-41-4, 100-42-5, 100-52-7, 10043-01-3, 10043-35-3	276

S1.4 Usage data organized by function, product, and product category

Table S4: Additive-product associations. These associations are pulled from Table S2 and reorganized by product. An abbreviated table is shown below. The full table can be found in Sheet S4 in Supporting Information S1 and at *https://github.com/sophievincoff/Plastic-Additives/blob/main/TableS4.csv*.

Product	Additives	Total Additives
(thin) sheet materials	122-62-3	1
accessories	24959-67-9,7439-92-1,7439-97-6,	6
accumulators	10108-64-2,10124-36-4,1306-19-	8
adhesive	100-42-5,100-51-6,10043-35-3,1	440
adhesive electronics	25038-59-9,7440-50-8	2
adhesives	101-68-8,117-84-0,118-82-1,1241	38
air tubes	117-81-7	1
alcohol-based cosmetics	131-56-6	1
animal collars	105-99-7	1
animal feed	128-37-0	1
anti-corrosion materials	26761-40-0	1
anti-fogging agents	26855-44-7,52497-24-2,68004-11	4
anti-fouling paints	26761-40-0	1
appliances	67774-32-7	1

Table S5: Additive-function associations. These associations are pulled from Table S2 and reorganized by function. An abbreviated table is shown below. The full table can be found in Sheet S5 in Supporting Information S1 and at *https://github.com/sophievincoff/Plastic-Additives/blob/main/TableS5.csv*.

Function	Additives	Total Additives
3D crosslinker	2451-62-9	1
accelerator	100-41-4,100-42-5,102-08-9,	19
acid scavenger	110-86-1,1309-42-8,1592-23-	8
acidifying agent	1336-21-6	1
activator	100-41-4,101-37-1,101-68-8,	16
additive	10043-35-3,10094-45-8,101-	179
adhesion promoter	142-82-5,1760-24-3,25068-3	12
adhesive	100-42-5,100-51-6,10043-35-	442
amine catalyst	100-74-3,280-57-9	2
anti-blocking agent	10094-45-8,110-31-6,112-84-	19
anti-foaming agent	102-71-6,104-76-7,108-88-3,	72
anti-fogging agent	111-03-5,1338-39-2,25496-7	10
anti-friction agent	112-80-1,128-37-0,1592-23-	30
anti-scratch agent	67701-03-5	1
anti-slip agent	14807-96-6,7440-66-6	2
anti-tack agent	10094-45-8,110-31-6,112-84-	17
anti-tack compound	57-11-4	1
antimicrobial	108-95-2,11096-42-7,111-30-	71
antioxidant	100-42-5,10039-33-5,10043-	376

Table S6: Additive-product category associations. Product strings were filtered into relevant product categories using both positive (*Positive Search Strings*) and negative search strings (*Negative Search Strings*). For example, "fragrances" (under *Products*) was included in "cosmetics" (*Products Category)* because of positive search string "fragr", but "toxic hazardous fragrances" was excluded from this category due to negative search string "toxic". This table can be found in Sheet S6 in Supporting Information S1 and at *https://github.com/sophievincoff/Plastic-Additives/blob/main/TableS6.csv*.

Product Category	Additives	Positive Search Strings	Negative Search Strings	Products
food	661	candy,cheese,cracker box,food,meat,plate,snack		candy bags,cheese packaging,cracker box covers,enclosures for food containers,food contact,food contact plastics coatings silicones,food contact plastics containing silicones,food packaging,food storage containers,food wrappers,food-contact products,food-contact wrapping,meat can covers,meat tray containers,plastic wraps for food storage,plates,snack packs
packaging (any)	364	bag,container,oilcloth,packaging,packing,packs,water proofing,wrap	bean bag filling	bags,blister packaging,blood storage bags,candy bags,cheese packaging,containers,dialysis bags,enclosures for food containers,food packaging,food storage containers,food wrappers,food-contact wrapping,green packing string,juice packs,manufacturing container metals,manufacturing containers,meat tray containers,oilcloths,packaging,packaging foam,packaging strings,packings,paper packaging,pharmaceutical packaging,plastic bags,plastic wraps for food storage,retail packaging materials,shrinkwrap,snack packs,soap packaging,soda bottle wrappers,soft packaging for toys,water bottle wrappers,water proofing materials,white packing string,wrapping films,yellow packing string
clothing	134	cloth,footwear,raincoat,rainwear,shoe,textil,water proofing	tablecloth,toy	childrens clothes,clothing,footwear,oilcloths,plastic print on clothing,plastic shoes,print on clothing,printing inks for clothing,raincoats,rainwear,shoes,sports shoes,textile fiber cover,textile inks,textiles,water proofing materials
drinking/water/fluids	128	cups,drink,juice,soda bottle,water,water bottle	pipes,water proofing,wrappers	childrens drinking bottles,cups,drinking water contact,drinkware,juice packs,soda bottle caps,soda bottles,water bottle caps,water bottles
furniture/household	114	bed,blinds,carpet,ceiling,cushion,decorat,door,floor,furnish,furniture,mattres,oilcloth,PVC flooring,roof,shower curtain,tablecloth,upholstery,vinyl flooring,wall	automotive,dental,upholstery for elv,water proofing	bedding,blinds,carpet back coating,carpet backing,cushions,decorative trims,doors,floor coverings,floor finishes,floor mats,floor tiles,furniture,furniture coatings,furniture upholstery,household furnishings,mattresses,oilcloths,plastisol roof,PVC flooring,roofing,shower curtains,tablecloths,under floor sealing compounds,upholstery,upholstery for furniture,vinyl floor tiles,vinyl flooring,vinyl wallpaper,wall covering,wall coverings,window frames,windows
building/construction	112	building material,cable,construction mater,flooring,gasket,insulation,pipe,piping		building material agents,building materials,cable flooring,cable insulation,cable wearer,cables,construction materials,downpipes,electrical cables,electrical insulation materials,flooring,flooring materials,gaskets,high-temperature cables,insulation,PVC flooring,sewage pipes,vinyl flooring,water pipes,wire flooring
electronics	91	computer,elec,pc,phone,tv,weee		adhesive electronics,casing for electronics,cellphones,computer casing,consumer electronics,electrical and electronic equipment (eee),electrical and electronic products,electrical cables,electrical insulation materials,electrical wire,electrical wire jackets,electronic components,electronic equipment,electronic toys,electronic waste,electronics,heat-resistant electrical cords,hydraulic fluid dielectric fluids in capacitors,low-price electronics,pc casing,personal computers,recycled weee plastic,recycled WEEE plastic,shredder residue from cases in electronics,shredder residue from mixed electronic plastics,tv,tv casing,WEEE
toys/games	69	bean bag,doll,game,play ball,playing,toy		bath toys,bean bag filling,childrens toys,dolls,electronic toys,game controllers,masquerade toys,plastic toys,play balls,soft packaging for toys,soft plastic toys,soft squeeze toys,toys,toys from textile
bottles	45	bottle		bottles,childrens drinking bottles,soda bottle caps,soda bottle wrappers,soda bottles,water bottle caps,water bottle wrappers,water bottles
children	44	child,kid		cd-disk for children,childcare articles,childrens clothes,childrens drinking bottles,childrens products,childrens toys,construction kits for children,stickers for children
automotives	29	car ,auto ,automobile,automotive		auto transmission lubricants,automobile parts,automotive coatings,automotive parts,automotive upholstery,automotives,automotives products,car fittings,car sealants,car seat stuffing,car seats,low-fogging automotive interiors
kitchen	26	blender,bowl,cook,kitchen		bowls,cookware,hand blenders,kitchen appliances
cosmetics	18	cosmetic,fragr,makeup,perfum	toxic	alcohol-based cosmetics,cosmetics,fragrance ingredients,fragrances,perfumes
medicine	16	blood,catheter,dental,dialysis,impregnation,medic,pharma		blood storage bags,dental cushions,dental fillings,dental sealants,dialysis bags,dialysis equipment,impregnation products,intravenous catheters,medical products,medical tubing,pharmaceutical packaging,pharmaceutical pills,pharmaceuticals,polysulfide dental impression materials
babies	5	baby,pacifier		baby changing mats,baby products,pacifier clips,pacifier coverage,pacifiers
pets	2	pet,pets	carpet	pet articles

S1.5: Identification of inconsistent chemical classifications

Table S7: Inconsistent chemical classifications between IARC and IRIS databases. This table can be found in Sheet S7 of Supporting Information S1 and at *https://github.com/sophievincoff/Plastic-Additives/blob/main/TableS7.csv*.

CAS Number	Chemical Name	IARC Group	IRIS_Assessment	IRIS_Weight-of-Evidence Characterization
78-87-5	1,2-Dichloropropane	1	Noncancer	No Classification
103-23-1	Di(2-ethylhexyl) adipate	3	Cancer	Possible Carcinogen
103-33-3	Azobenzene	3	Cancer	Probable Carcinogen
1163-19-5	Decabromodiphenyl oxide	3	Cancer	Suggestive Evidence of Carcinogenic Potential
79-00-5	1,1,2-Trichloroethane	3	Cancer	Possible Carcinogen
100-42-5	Styrene	2A	Noncancer	No Classification
1309-64-4	Antimony trioxide	2A	Noncancer	No Classification
593-60-2	Vinyl bromide	2A	Noncancer	No Classification
68-12-2	N,N-Dimethylformamide	2A	Noncancer	No Classification
71-55-6	1,1,1-Trichloroethane	2A	Noncancer	No Classification
100-41-4	Ethylbenzene	2B	Noncancer	No Classification
106-46-7	para-Dichlorobenzene	2B	Noncancer	No Classification
106-47-8	para-Chloroaniline	2B	Noncancer	No Classification
106-88-7	1,2-Epoxybutane	2B	Noncancer	No Classification
109-99-9	Tetrahydrofuran	2B	Noncancer	No Classification
110-86-1	Pyridine	2B	Noncancer	No Classification
121-14-2	2,4-Dinitrotoluene	2B	Noncancer	No Classification
122-39-4	Diphenylamine	2B	Noncancer	No Classification
1314-62-1	Vanadium pentoxide	2B	Noncancer	No Classification
2385-85-5	Mirex	2B	Noncancer	No Classification
26471-62-5	Toluene diisocyanates	2B	Noncancer	No Classification
75-35-4	Vinylidene chloride	2B	Noncancer	No Classification
765-34-4	Glycidaldehyde	2B	Noncancer	No Classification
79-46-9	2-Nitropropane	2B	Noncancer	No Classification
91-20-3	Naphthalene	2B	Noncancer	No Classification
98-82-8	Cumene	2B	Noncancer	No Classification

S1.6: Identifying significant data coverage differences across IARC categories

Paired t-tests were performed between each pair of IARC categories (*e.g.,* 1 vs. 2A, 1 vs. 2B, 1 vs. 3, 1 vs. unclassified) using the scipy.stats package in Python, to determine any significant differences in data coverage. To assemble lists for comparison, the additive database in Table S2 was filtered by each IARC category, and the *Total Polymers*, *Total Products*, *Total Functions*, and *Total PMIDs on Gene Interactions* columns were pulled. The resulting p-values for each comparison are shown below. In row 1 of Table S8, IARC category 1 and 2A have significant differences in their *Total Functions* columns ($p = 0.045$) and *Total PMIDs on Gene Interactions* ($p = 0.018$) columns, meaning there are significant differences in the number of functions associated with Group 1 chemicals vs. Group 2A chemicals, and the number of publications on gene interactions concerning Group 1 chemicals vs. Group 2A chemicals. These results were used to determine whether some IARC groups were more well-studied than others.

Table S8: Significant differences in data coverage between additives in IARC Group 1, 2A, 2B, 3, and unclassified. *P-values* are determined by paired t-test. This table is available in Sheet S8 of Supporting Information S1 and at *https://github.com/sophievincoff/Plastic-Additives/blob/main/TableS8.csv*.

IARC Categories Compared					p-value			
					Total Polymer Types	Total Products	Total Functions	Total PMIDs on Gene Interactions
1	2A				0.608	0.483	0.045	0.018
1		2B			0.524	0.507	0.021	0.000
1			3		0.203	0.481	0.047	0.001
1				Unclassified	0.001	0.068	0.000	0.000
	2A	2B			0.923	0.968	0.695	0.371
	2A		3		0.416	0.684	0.632	0.737
	2A			Unclassified	0.003	0.432	0.019	0.105
		2B	3		0.346	0.627	0.846	0.235
		2B		Unclassified	0.000	0.245	0.000	0.094
			3	Unclassified	0.003	0.027	0.000	0.000

(shaded) $p < 0.05$
